# Study on the extension law of fractures in shale reservoirs under impact loads

**DOI:** 10.1371/journal.pone.0328782

**Published:** 2025-07-22

**Authors:** Wei Li, Zhuolun Li, Ying Li, Huan Zhao, Shuangyang Wang, Xingsheng Xu, Jiahao Kong

**Affiliations:** 1 Northeast Petroleum University, College of Petroleum Engineering, Daqing, China; 2 State Key Laboratory of Continental Shale Oil, Daqing, China; 3 National Engineering Research Center of Oil & Gas Drilling and Completion Technology, Beijing, China; 4 Heilongjiang Provincial Key Laboratory of Oil and Gas Reservoir Stimulation, Daqing, China; 5 PetroChina Tarim Oilfield Company, Korla, China; China University of Petroleum East China - Qingdao Campus, CHINA

## Abstract

Fracturing technology is an important technique in the development of shale reservoirs. Compared with conventional hydraulic fracturing, explosive fracturing technology has the advantages of low cost and environmental protection. Relevant research results have shown that this technology can effectively improve the efficiency of shale oil and gas extraction. To further reveal the mechanism of reservoir fracture propagation under explosive fracturing, this paper conducted experimental research on the propagation law of complex fractures in shale reservoirs under impact load. The dynamic elastic modulus of shale samples was tested by using the Split Hopkinson Pressure Bar (SHPB) test system. A finite element model was established by using LS-DYNA software. Based on test results, the fracture propagation process was simulated under different impact loads, interlayer spacing, and fracture distribution conditions. The results indicate that the original crack zone of layered reservoirs is more prone to induce stress, which is beneficial for reservoir transformation in the near wellbore area, while explosive fracturing. The increase in interlayer spacing is beneficial for the expansion of the main crack, which can improve reservoir connectivity. As the impact load increases, the main cracks have better connectivity. This study can provide a theoretical basis for optimizing fracturing parameters and designing fracturing schemes, which is of great significance for the promotion of explosive fracturing technology and the efficient and environmentally friendly development of shale oil.

## 1. Introduction

With the Chinese economy moving towards high-quality development, the demand for oil, especially natural gas, is rapidly increasing. Shale oil and gas resources have become the mainstay of crude oil production growth. The exploration and development of shale oil and gas is of great significance for ensuring China’s energy security, improving its energy structure, and promoting technological progress in China’s oil and gas industry [1–4]. Due to the low porosity and permeability of shale reservoirs [5], fracturing technology has become a key technology in the development process of shale reservoirs [6–8]. The conventional hydraulic fracturing process consumes a large amount of water, making it difficult to achieve economic benefits and posing a threat to groundwater resources. Therefore, to improve the green and efficient exploitation level of oil and gas resources, an anhydrous reservoir stimulation technique with high peak pressure needs to be developed, such as explosive fracturing technology [9–11].

In response to the problem of poor hydraulic fracturing performance in oil fields with poor connectivity, American technicians proposed explosive fracturing technology in the 1960s [12], which is a more convenient and low-cost transformation method than hydraulic fracturing. Early research focused on experiments to verify the fracturing effect of explosive fracturing. Experimental study by Kutter and Fairhurst [13] explored the roles played by stress waves and gas pressure during deflagration fracture, noting that stress waves are primarily responsible for generating numerous fractures, while gas pressure is the main driving force for continued fracture expansion. The study of Sandia National Laboratories and Lawrence Livermore Laboratory in the United States confirms the important role of the ignition blast loading rate and dynamic shock on fracture initiation, proving the wedging effect of the ignition blast on the fractures [14].

The cracking process under stress wave loading is considered the crucial stage, as the subsequent fragmentation due to continuing penetration of the explosion gases is largely guided by this initial fractured state. Therefore, it is particularly important to study the influence of blasting characteristics on crack propagation. Zhu et al. [15] analyzed the fracture mechanism of the rock under the action of dynamic loads, and pointed out that there were many factors which affected rock fracturing, such as coupling medium, conﬁnement, boundary condition, initiation location in an explosive column, and air ducking. And it is believed that the coupling medium and the location of the explosion have a particularly significant effect on rock fracture. Balakrishnan et al. [16] carried out a numerical simulation study regarding the explosion characteristics of explosives in homogeneous reservoirs, estimated the shock overpressure and total impulse at different radial locations, and compared the different explosives, including Nitromethane, Trinitrotoluene, and High-Melting Explosive. Finally, a method for designing explosives based on explosive energy was proposed. An et al. [17] investigated the dynamic fracture phenomenon of rocks under the effect of blast impact, and analyzed the effects of various properties of explosives and rock on the rock fracture process. Wan et al. [18] proposed a shale gas completion fracturing technology using fuel-air explosives (FAE)to control combustion explosion, and simulated the explosion effect. The simulation of FAE burning explosion included the relationship between explosion pressure and charge mass, explosion heat, ideal coefficient, inertia coefficient, and initial pressure. The numerical simulation shows that the explosion pressure is positively correlated with the charge mass.

With the development of numerical simulation technology and the application of commercial simulation software, more and more scholars are using simulation software to analyze the effect of explosive fracturing on different rocks. Hao et al. [19,20] established an equivalent simulation method for the explosion of explosives in Granite and conducted numerical simulation analysis on the propagation of explosive stress waves and estimation of rock damage areas. Galybin et al. [21] studied the initiation and propagation of cracks in elastic plates with initial straight cracks, and concluded that stress fluctuations change the local stress equilibrium field, which in turn affects the fracture extension path. Hajibagherpour et al. [22] numerically simulated the mechanism of rock fragmentation due to blast-induced shock waves in a single blasthole by a two-dimensional discrete element code. Compared with the corresponding experimental results, they concluded that the proposed numerical model could be effectively used for the simulation of the crack propagation process around a Barre granite blasthole. Wang et al. [23] presented a hybrid continuum-discontinuous method considering the actual breakage of rocks and investigated the fracture mechanisms of high-pressure gas explosion fracturing, which considered Barre granite fracture and exfoliation due to dynamic blast impacts. Yue et al. [24] proposed a rock blasting model based on the continuum-discontinuous element method, which successfully simulated the whole rock blasting process.

The above-mentioned research objects are mostly granite, sandstone, and concrete. The biggest feature of shale compared to granite and sandstone is its natural complex joint structure. In the study on dynamic mechanical laws in jointed rock masses, the main loading methods concerned include drop weight impact (DWI), split Hopkinson pressure bar (SHPB), and blasting tests. Among the above-mentioned loading methods, the SHPB test can effectively simulate dynamic disturbances such as rock bursts and earthquakes [25]. The prefabrication of joints in rock mass is a common method for studying shale. Zhou and Gu [26] conducted an SHPB test on granite specimens with various joint configurations to figure out the mechanical properties of persistently fractured granite under impact loading with varying rates. In terms of blasting tests, digital laser dynamic caustics (DLDC) have been widely applied. Shen et al. [27] conducted blasting experiments on red sandstone, which prefabricated parallel joints with different spacings on the surface and monitored the crack propagation speed and length. Then tracked the propagation process of blasting cracks and the evolution process of blasting stress waves in parallel joints with the discrete element software PFC.

With the further development of shale oil and gas resource development technology, research on the explosive fracturing of shale has begun to increase in recent years. Guo et al. [28] proved that, compared with hydraulic fracturing, the direction of fracture formed by explosive fracturing was independent of the formation stress. Wang et al. [29] studied the samples which were taken from the outcrop of shale from the Ordovician Wufeng Formation-Silurian Longmaxi Formation in Changning County, Sichuan Province. Then constructed a multi-physics field coupling numerical model of deflagration fracturing considering the synergistic effects of stress impact and gas wedging based on the continuum-discontinuous element method, and numerical studies of staged methane deflagration fracturing in horizontal wells under different factors were performed. Wang et al. [30] established a numerical simulation model of methane explosion fracturing in the in-situ shale reservoir, and analyzed the effects of explosive loading, in situ stress, and stress difference on the fracturing efficiency. Wang et al. [31] fractured prefabricated perforated shale samples with parallel and vertical bedding under ﬁve distinct explosion loads using a methane in-situ explosion fracturing experimental setup. The research results confirm that the explosion load and bedding direction have a significant impact on the propagation mode of cracks.

Although explosive fracturing technology has been studied and applied in the petroleum industry for more than 30 years, relevant research results have shown that this technology can effectively improve the efficiency of shale oil and gas extraction. However, the complex reservoir conditions of shale oil and gas and the mechanism of reservoir fracture propagation are not yet very clear, which cannot effectively guide the design of explosive fracturing, resulting in the inability to apply explosive fracturing technology on a large scale in China.

In order to clarify the propagation law of shale reservoir fractures under impact loads of explosive fracturing, the dynamic elastic modulus of shale samples was tested by using the Split Hopkinson Pressure Bar (SHPB) test system, and a finite element model was established by using LS-DYNA software. Based on test results, a study on the fracture propagation law of shale reservoirs under impact loads was conducted by using the finite element method. Then, the fracture propagation process was simulated under different impact loads, interlayer spacing, and fracture distribution conditions. This study can provide a theoretical basis for optimizing fracturing parameters and designing fracturing schemes, which is of great significance for the promotion of explosive fracturing technology and the efficient and environmentally friendly development of shale oil.

## 2. Rock mechanics parameter test

### 2.1. The split hopkinson pressure bar test system

The Split Hopkinson Pressure Bar (SHPB) test system has been widely used in the field of scientific research, which is a test method used to measure the dynamic mechanical properties of materials at high strain rates [32,33]. The system loads a sample by applying a shock wave and measures its stress-strain response, making it particularly advantageous in rock burst prediction, rock property exploration, stress disturbance studies, and dynamic impact simulations. [34,35].

**Fig 1:**
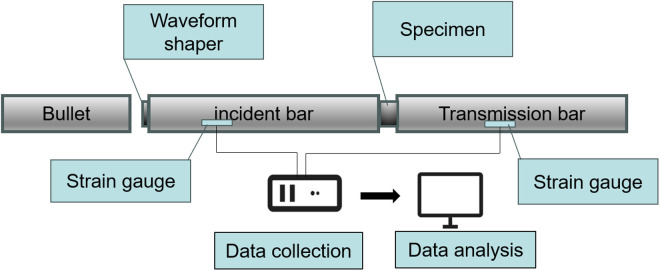
Schematic diagram of the SHPB test system.

The SHPB test system mainly consists of a bullet, a waveform shaper, an incident bar, a specimen, a transmission bar, strain gauges, a data collection system, and a data analysis system. The schematic diagram of the test device is shown in Fig 1. In the SHPB setups, the bullet moves forward under the push of high-pressure gas and reaches a certain speed. After passing through a velocity measurement system, the bullet impacts the incident bar, generating a stress wave that travels along the bar. When the stress wave reaches the specimen, it partially reflects and transmits. The transmitted wave passes through the specimen and similarly reflects and transmits at the interface between the specimen and the transmission bar, ultimately reaching stress equilibrium. The strain gauges on the bars capture the stress wave propagation, and the data is recorded and processed by a high-speed dynamic strain gauge, yielding strain-time histories of the incident, reflected, and transmitted waves [36]. Then use the three-wave method to determine the changes in stress, strain, and strain rate [37].

The length of both the incident bar and the transmission bar is 4500 mm. The diameter of each rod is 80 millimeters, the elastic modulus is 206 GPa, the wave velocity is 5100m/s, and the density is 7.81g/cm^3^. The shale specimen is placed between the incident bar and the transmission bar to maintain a one-dimensional plane.

### 2.2. Materials

The shale samples studied in this article were collected from the GY1 well in the middle upper part of the Gulong depression in the central depression area of the Songliao Basin, with a sampling depth of 2469.99-2497.41m. The well is located at the center of the Qijia depression and Gulong depression, with a relatively thick formation thickness. The Qingshankou Formation has a thickness of over 400 meters and is a semi deep lake deep lake sediment with a relatively stable sedimentary environment [38]. The main lithology is dark gray mudstone. Grind 50-60g of the sample and divide it into two groups for mineral composition analysis. The TD-3500 X-ray diffractometer produced by Dandong Tongda Technology Co., Ltd. is used for mineral composition analysis in this test. The shale mineral composition parameters are shown in Table 1.

**Table 1:** Shale mineral composition parameters.

sample number	Mineral composition (%)							
	Clay	quartz	plagioclase	siderite	hematite	iron pyrite	gypsum	dolomitic
1#	37	21.6	12.2	1.3	7.4	11.5	3.7	5.3
2#	36.3	22.1	17.6	1.6	6.8	7.7	3.5	4.4
average value	36.65	21.85	14.9	1.45	7.1	9.6	3.6	4.85

The mineral composition of the sample consists of clay minerals, quartz, and plagioclase; Among them, the mass fraction of brittle minerals [39] (quartz, plagioclase, pyrite, dolomite) reaches 51.2%, the clay component is 36.65%, and other minerals (such as hematite, siderite, and gypsum) reach 12.15%. Based on the mineral composition, it is preliminarily determined that the shale samples are highly brittle with high strength.

**Fig 2:**
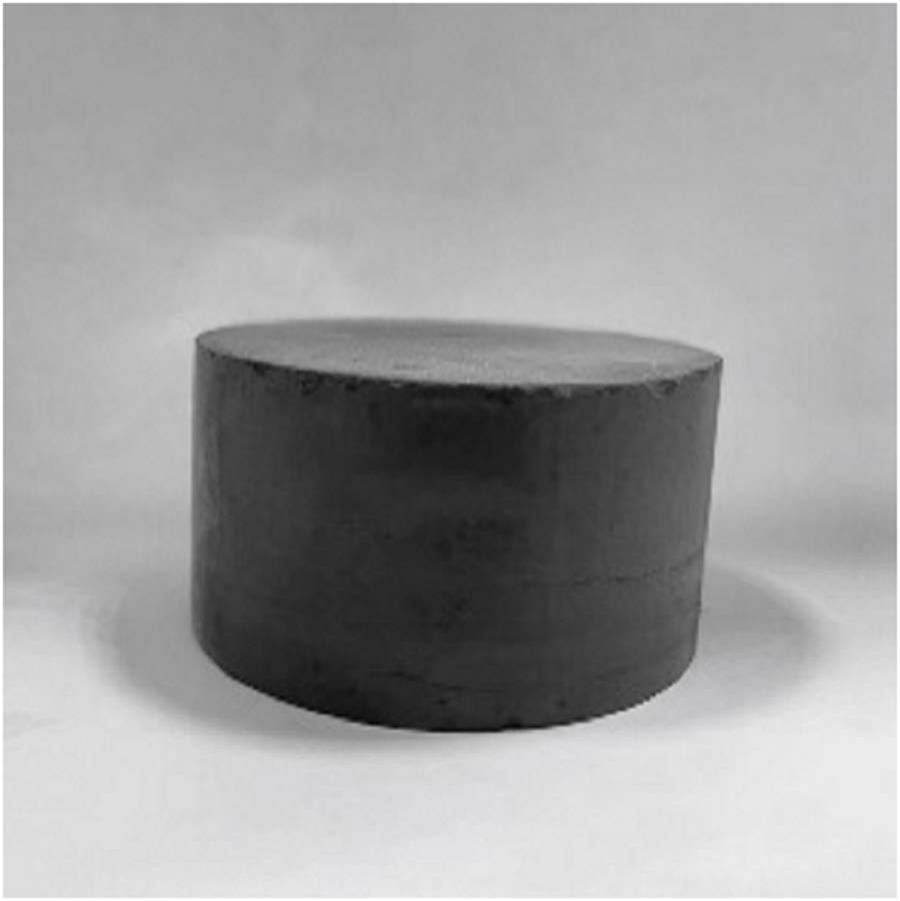
Specimen.

The shale specimen is drilled from the shale sample. The specimen is cylindrical with a size of 50 mm × 25 mm and a length to diameter ratio of 1:2(Fig 2). The processing of the specimen follows the ISRM standard specification: its flatness is within 0.05 mm, the diameter error along the height of the specimen is not greater than 0.3 mm, the sample end face is perpendicular to the centerline, and the deviation angle is less than 0.25°.

### 2.3. Test scheme

To study the dynamic mechanical properties of shale under impact loading, multiple pre-tests were conducted, and the final designed test strain rate was between 10 and 500/s^-1^. To ensure effective testing of the mechanical properties of shale, data from impact tests on shale under various strain rates were analyzed to ensure the accuracy of the mechanical property measurements for each strain rate. During the test process, the damage morphology of shale specimens under different strain rates was observed by adjusting the impact air pressure [40].

The impact air pressures were 0.06MPa, 0.08MPa, and 0.1MPa, respectively, corresponding to test impact velocities of 3.386m/s, 4.288m/s, and 5.126m/s. Three specimens were impacted under each impact air pressure group to exclude the single specificity of the specimen samples.

Before each impact test, grease was applied to both end surfaces of the specimens, which was used to attenuate the frictional effect between the specimens and the rod [41]. After each specimen test is completed, collect the crushed blocks of the specimens and observe their external characteristics.

### 2.4. Test data calculation method

The SHPB test data in this article were calculated using the three-wave method to determine the changes in stress, strain, and strain rate, as shown in equations (1) – (3) [42].


$$\sigma (t)=\frac{A_{e}E_{e}}{A_{s}}\varepsilon_{T}(t)$$ (1)

$$\varepsilon (t)=\frac{{-2C}_{e}}{L_{s}}\int_{0}^{t}\varepsilon_{R}(t)d\tau$$ (2)

$$\overset{.}{\varepsilon }(t)=\frac{{-2C}_{e}}{L_{s}}\varepsilon_{R}$$ (3)

Where $\varepsilon_{I}(t)$, $\varepsilon_{R}(t)$, $\varepsilon_{T}(t)$represent the strains produced by the incident wave, reflected wave, and transmitted wave, respectively. $A_{e}$, $E_{e}$ are the elastic modulus and cross-sectional area of the bars, $C_{e}$ is the propagation velocity of the stress wave in the bars, $A_{s}$, $L_{s}$ are the initial cross-sectional area and length of the sample, respectively.

Considering stress equilibrium, we obtain Equation (4):


$$\varepsilon_{I}(t)+\varepsilon_{R}(t)=\varepsilon_{T}(t)$$ (4)

### 2.5. Test results and stress balance analysis verification

**Fig 3:**
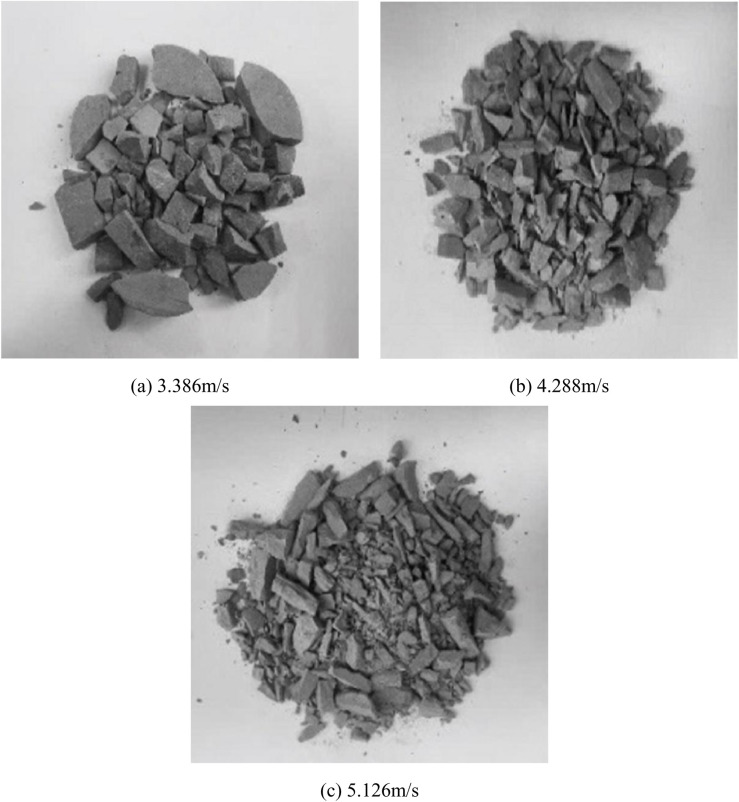
Graph of test results. (a) impact velocity of 3.386m/s, (b) impact velocity of 4.288m/s, (c) impact velocity of 5.126m/s.

**Fig 4:**
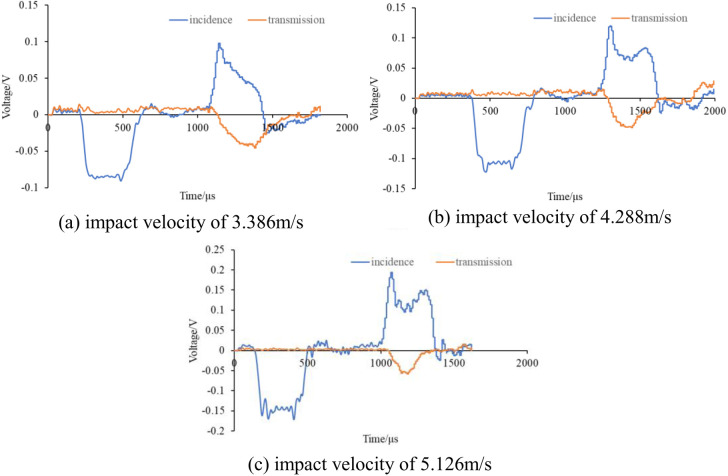
Impact waveform diagram. (a) 3.386m/s, (b) 4.288m/s, (c) 5.126m/s.

The impact air pressures were 0.06MPa, 0.08MPa, and 0.1MPa, respectively, corresponding to test impact velocities of 3.386m/s, 4.288m/s, and 5.126m/s. The dynamic loading tests were carried out on the above three groups of specimens under different impact loads, and the dynamic stress-strain curves of the same group of specimens were relatively close. Therefore, the test data of one specimen in each group were selected for comparative analysis. Fig 3 illustrates the damage morphology observed in the test; the impact waveforms of the specimens are shown in Fig 4.

As shown in Fig 3, at an impact velocity of 3.386 m/s, the specimen was completely destroyed, with the body split into large pieces; at 4.288 m/s, the specimen was completely split into small pieces; at 5.126 m/s, the specimen was completely crushed and destroyed, with the body completely split into powder and smaller pieces.

**Fig 5:**
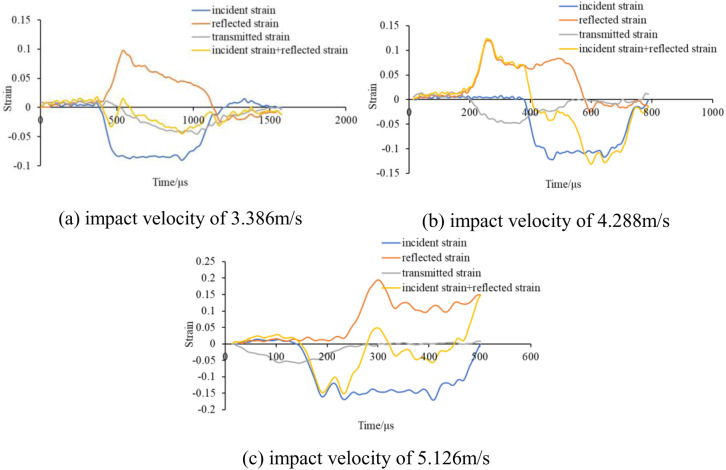
Dynamic strain equilibrium curves of specimens. (a) impact velocity of 3.386m/s, (b) impact velocity of 4.288m/s, (c) impact velocity of 5.126m/s.

The three-wave method was used to process the data from the rock mechanics test results. The calculation formulas are shown in equations (1)- (3). Equation (4) can be used to verify that the specimen has reached stress equilibrium in a dynamic triaxial test. When the duration of the stress wave is 400 μs, the dynamic strain equilibrium curves of the specimen are shown in Fig 5.

Fig 5 shows that the sum of incident strain and reflected strain is basically equal to the transmitted wave strain, and the stress balance condition at both ends of the specimen can be satisfied, therefore, the test results are reliable.

### 2.6. Analysis of SHPB test results

**Fig 6:**
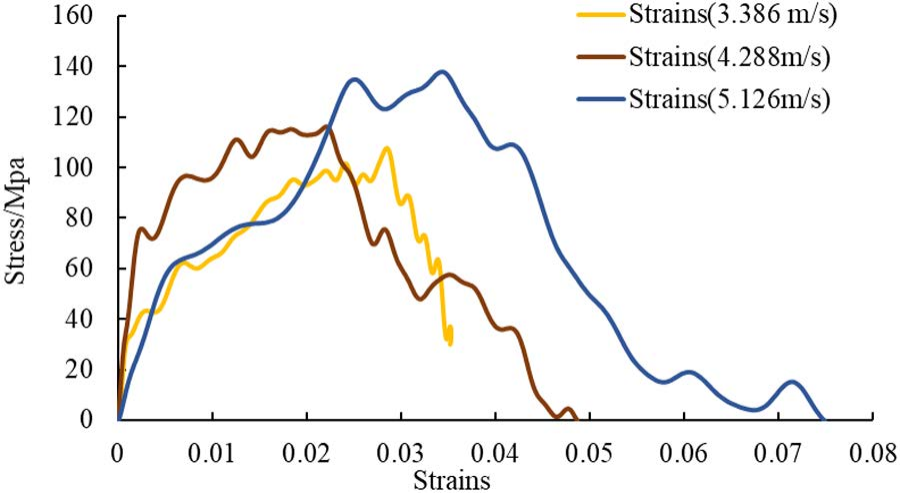
Stress-strain curves of shale specimens under four different impact loads.

The dynamic stress-strain curves of shale show different deformation characteristics under impact loading. Fig 6 depicts the variation of stress-strain curves of laminated shale specimens under different impact velocities.

The dynamic stress-strain curve can be divided into three main stages, as shown in Fig 6: an initial linear phase, a yielding phase, and an unloading phase. Before the specimen reaches the yield state, the tangent slope of the stress-strain curve increases and then decreases; then the stress continues to increase, and the yield plateau rises significantly; finally, a sharp decrease in stress occurs during the unloading stage. The dynamic mechanical parameters of shale exhibit considerable variability with the change in strain rate. The increase in impact load increased to the dynamic yield point of the shale specimens, while the dynamic fracture toughness and dynamic plasticity showed a decreasing trend, and the destructive strain of shale increased significantly, particularly affecting the strength characteristics and elastic modulus. The shale elastic modulus was calculated to be an average of 4.42 GPa.

## 3. Numerical simulation model

### 3.1. Finite element model of SHPB test

**Fig 7:**
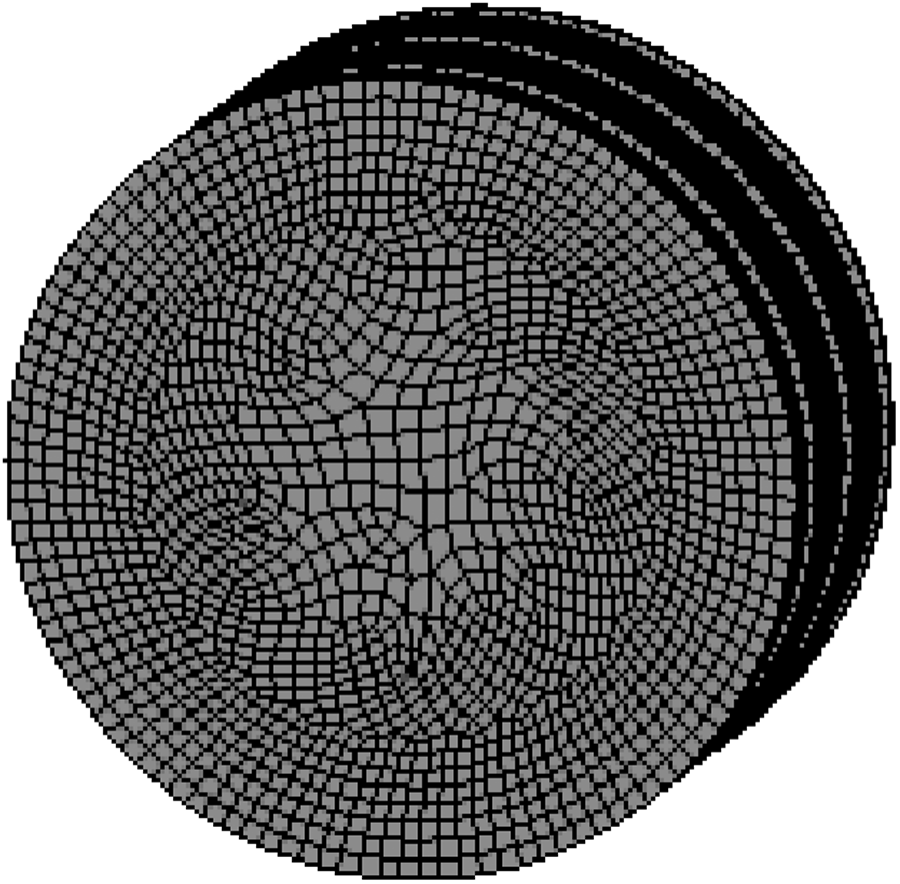
Shale rock model.

Based on the SPHB dynamic testing theory, ANSYS/LS-DYNA is applied to model shale rocks and compression bars. The shale rock model adopts an 8-node solid element, Solid164, to establish a three-dimensional cylindrical solid, with a model size of 50 mm in diameter and 25 mm in height. The numerical models in this article are all based on the g-cm-µs unit system, with an overall grid division size of 10 mm. The mesh division of the model is shown in Fig 7.

**Fig 8:**
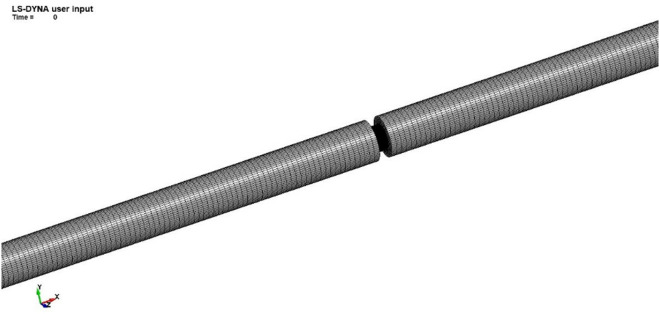
Bar model.

The bar model adopts an 8-node solid element, Solid164, to establish a three-dimensional cylindrical solid. The model size includes an incident rod with a length of 4500 mm and a transmission rod with a length of 4500 mm, both with a diameter of 80 mm. Each node contains 3 degrees of freedom, which can be used to effectively calculate the elastic response of the bar. The mesh division of the model is shown in Fig 8.

(1) Material Model

The material model of the compression rod is described using the keyword * MAT_SLATSIC, while the material model of the rock is described using the HJC model. The HJC model is a constitutive model with damage proposed by Holmquist et al. [43], which is originally developed for concrete under impact loading, and has been extended to rock material in recent years [44]. The HJC model considers the effects of large strain, high pressure, high strain rate, and damage on the mechanical properties of materials, and can accurately describe the failure process of rocks under strong dynamic loads [45].

The material parameters of the HJC model are obtained based on the results of experiments or by fitting experimental data [46]. Rock Material Model (HJC) parameters: density = 2.6g/cm^3^, A  = 0.79, B = 1.6, N = 0.61, C = 0.007 [47], standardized maximum strength valueSFMAX = 7.0, reference strain rate$\varepsilon =10^{-5}s^{-1}$, $D_{1}$ = 0.04 $D_{2}$ = 1.0, the cumulative plastic strain value before rock cracking EFMIN = 0.01 [48], crushing pressure$p_{c}$ = 0.04GPa, crushing strain$u_{c}$ = 0.001, $k_{1}$ = 85GPa, $k_{2}$ = −171GPa, $k_{3}$ = 208GPa, pore compaction pressure$p_{l}$ = 1.0GPa, rock porosity$u_{l}$ = 0.1 [49].

(2) Application of Load

Define the incident wave using the keyword * DEFINE_CURVE, and load the explosive shock incident wave at the end of the incident rod using the keyword * LOAD SEGMET SET to complete the application of the load.

(3) Determination of boundary conditions

The boundary conditions of this model are implemented using the keyword * BOUNDAR_SPC_SET to constrain the normal direction, and the keyword * BOUNDARYD_NON_REFELECTION is used to set the non-free surface of the model as a non-reflective boundary, absorbing expansion waves to simulate a semi-infinite medium.

(4) Hourglass control

Adjust the hourglass energy by adding the keyword * Control_fourglass, and adjust and control the quadratic and linear coefficients by adding the keyword * Control_fulk_viscosity to complete the process of increasing the volume viscosity in the material model.

In the simulation, the keyword * MATOAD_SROSION is used to control the failure of the specimen. Currently, commonly used failure criteria include maximum principal stress, maximum principal strain, maximum tensile stress, and maximum shear strain. Through the debugging of simulation parameters, it has been found that selecting appropriate values for maximum principal strain and maximum principal stress can achieve good simulation results. Therefore, this article adopts the maximum principal strain and maximum principal stress failure criteria, that is, if the failure strain of shale material exceeds the maximum principal strain or maximum principal stress, the element will fail.

In summary, the keywords for the contact type between the pressure rod and shale samples in this article are * CONTACT-ERODING_SURFACE_TO-SURFACE. The contact algorithm adopts the most widely used symmetric penalty function method, as it can effectively avoid the hourglass phenomenon. Secondly, LS-DYNA checks whether there is node penetration at each time step. When node penetration is detected, an interface contact force similar to a normal spring is set on the target contact surface, which, to some extent, ensures the stability of numerical calculations and the correctness of calculation results.

### 3.2. Validation of Numerical Model

**Fig 9:**
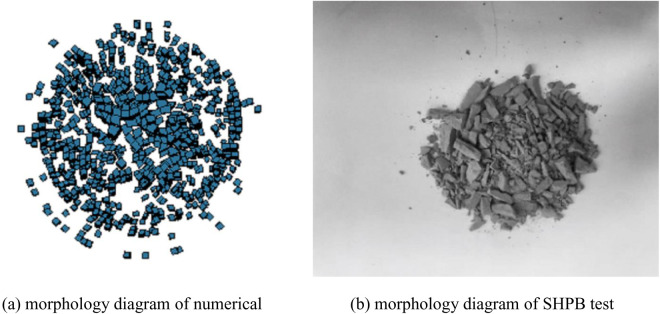
Simulation and test fragmentation morphology diagram. (a) morphology diagram of numerical simulation, (b) morphology diagram of SHPB test.

To validate the reliability of the numerical model, the shale fracture morphology diagram obtained from numerical simulation with an impact velocity of 5.126m/s is compared with the fracture morphology obtained from the SHPB test, as shown in Fig 9, indicating a high degree of consistency between them.

### 3.3. Shale reservoir fracture expansion model under impact loads

**Fig 10:**
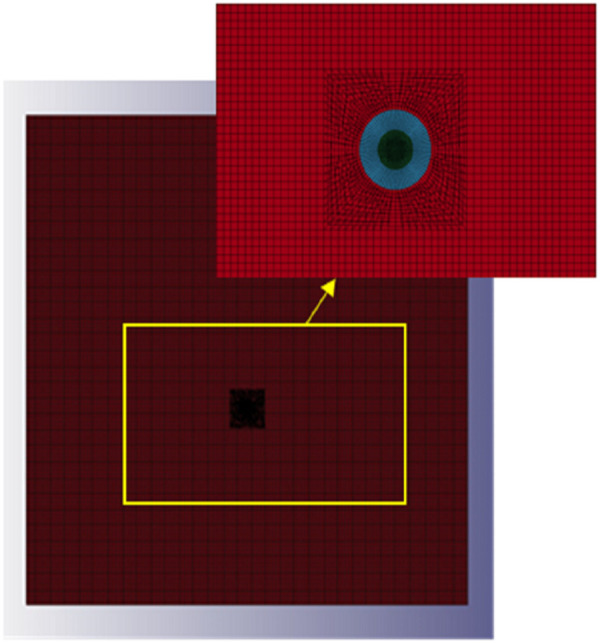
Explosive Model Diagram.

Under the action of explosive impact load, rock fracture primarily occurs when the impact stress exceeds its strength limit. Shale reservoirs exhibit strong non-homogeneity and anisotropy, making them very complex in practical research. To study the influence of layering as a single factor variable and avoid the influence of other factors, the model material has been simplified. In shale reservoirs, the extension region of a fracture is considered as a homogeneous and isotropic theoretical model. The finite element model consists of three parts: air, explosives, and the shale reservoir, all of which use 8-node solid elements Solid164. In this model, the shale reservoir applies the single-point integration algorithm of the Lagrange column type, which is favorable for large deformation situations and ensures stable and fast overall computation; for air and explosives, Eulerian grids are used for modeling, and the cell algorithms employ the multi-material fluid-solid coupling algorithm. The explosive cells are designated for the shale reservoir. A denser grid division is used, allowing the system to focus on calculating the explosive unit during the computation process, which helps in saving calculation time and improving calculation accuracy [50]. Uncoupled charges are used for central point detonation, with the explosives located at the center of the shale reservoir (Fig 10). Non-reflective boundary conditions are applied to the air edges, and the model is created using the cm-g-μs unit system.

(1) Finite element model of homogeneous reservoirs

**Fig 11:**
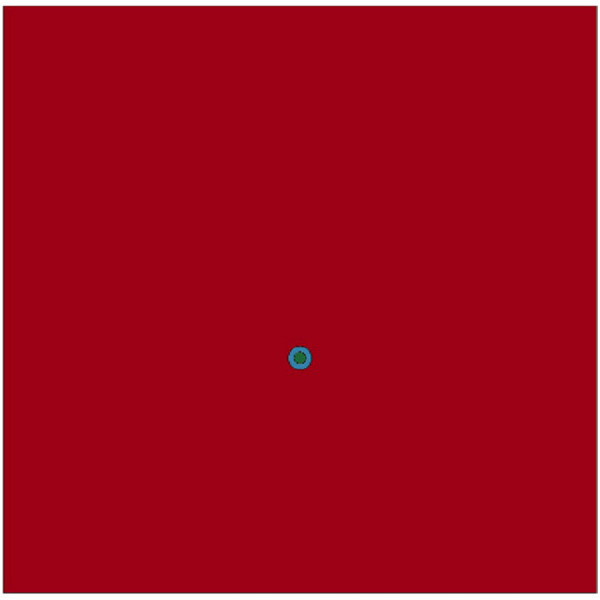
Finite element model diagram for homogeneous reservoirs.

Based on the theory of rock rupture and expansion in explosively fractured reservoirs, a finite element model was established by ANSYS/LS-DYNA software, with an overall mesh size of 1 cm. The finite element model is shown in Fig 11.

(2) Finite element model of reservoirs with a single layer

**Fig 12:**
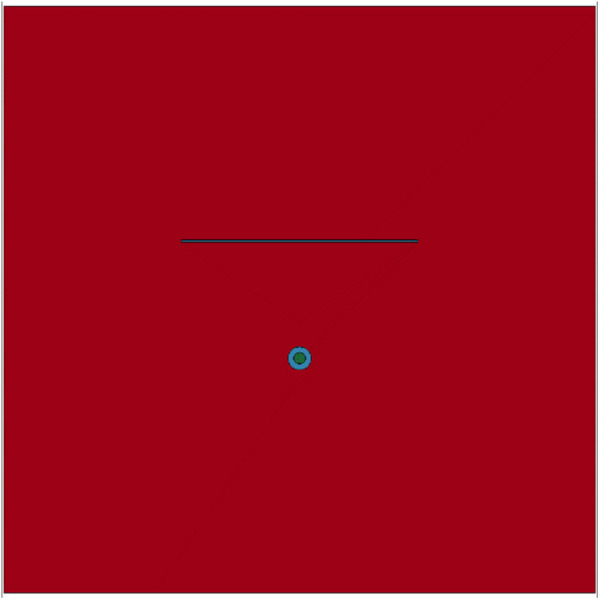
Finite element model of reservoirs with a single layer.

The impact load generated by explosive fracturing has a transformative effect on the fractures and pores of layered reservoirs. And as the reservoir bedding and microcracks become more developed, the transformation effect becomes more pronounced [51]. Shale reservoirs are layered. Based on the finite element model of homogeneous reservoirs, a fracture bedding plane is inserted to establish a finite element model of reservoirs under the impact load with a single layer. The bedding length is 100 cm, located 50 cm on one side of the borehole. The finite element model is shown in Fig 12. Design the explosion impact load as a variable parameter, and use other parameters as constant values for simulation analysis. By comparing the fracturing effects of homogeneous reservoirs and single layer reservoirs, the influence of single layer distribution on the formation of complex fractures can be analyzed.

(3) Finite element model of reservoirs with multiple layers

**Fig 13:**
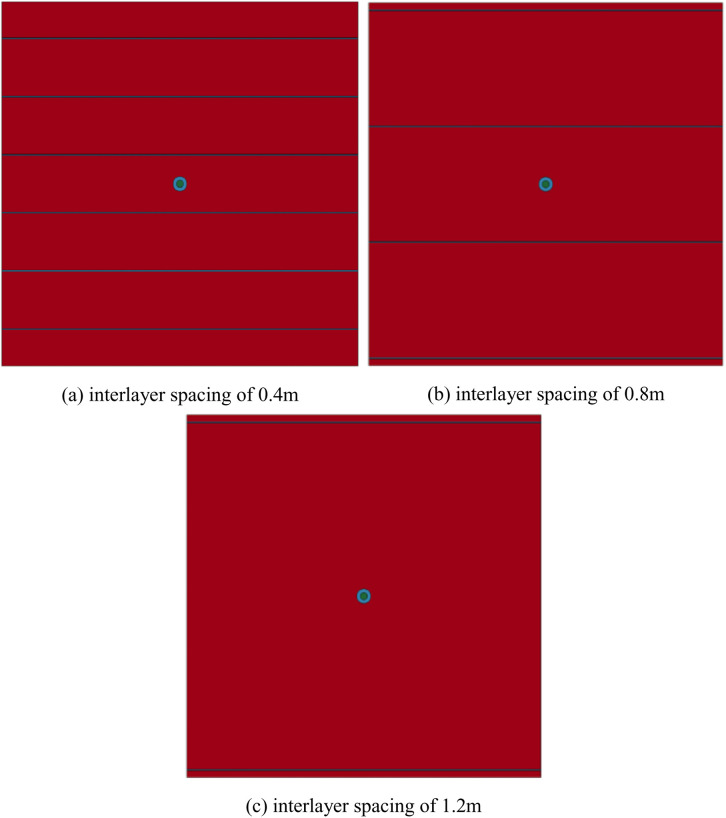
Finite element model of reservoirs with multiple layers. (a) interlayer spacing of 0.4m, (b) interlayer spacing of 0.8m, (c) interlayer spacing of 1.2m.

Under the impact load, the reservoir with multiple layers is more prone to forming complex fractures [52]. The degree of bedding development has a significant impact on the initiation and propagation of explosive fracturing. It is particularly important to explore the fracture propagation and fracture penetration between different interlayer distances under explosive impact loads through numerical simulations. Therefore, a finite element model is established for reservoirs with multiple layers. Based on the homogeneous reservoir model, three layered reservoir models with interlayer spacing of 0.4m, 0.8m, and 1.2m were set up as shown in Fig 13.

(4) Material models and equations of state

After completing the establishment of the numerical model in ANSYS, keywords are used to assign material properties to rocks and air, among others, and the model parameters are shown below. The material model of the rock and its adoption in this paper are described in Section 2.2.

The *MAT_NULL keyword is used to characterize the air. Specific information on the materials involved and the parameters of the equation of state can be found in Table 2.

**Table 2:** Air modelling parameters.

parametric	densities/(kg/m^3^)	Pc/MPa
worth	1.29	0

The explosive explosion process generates a strong impact pressure. As the volume of the blast product continuously expands, the pressure on the gun hole also changes. For the initial impact pressure generated by the explosion, the explosives model uses the LS-DYNA system’s embedded model, specifically the *MAT_HIGH_EXPLOSIVE_BURN model. Then, relevant control parameters are entered, including explosive density, explosive velocity, and blast C-J pressure, among other parameters. The software can solve the explosion process autonomously. The model describes the continuous expansion of the blast shock pressure with the explosive gas and its subsequent decay. The decay process can be described using the JWL equation of state [53]:


$$P=A(1-\frac{\omega }{R_{1}V})e^{-R_{1}V}+B(1-\frac{\omega }{R_{2}V})e^{-R_{2}V}+\frac{\omega E_{0}}{V}$$ (5)

Where: P represents the pressure of the products of the explosion; V represents the relative volume of the products of the explosion; $E_{0}$ is the initial internal energy of the explosive, the rest of the parameters are related to the control equation.

(5) Determination of boundary conditions

The boundary conditions of this model use the keyword *BOUNDARY_SPC_SET to achieve normal constraints. The keyword *BOUNDARY_NON_REFLECTING is set to the non-free surface of the model for the non-reflective boundary, absorbing shear waves and expansion waves, used to simulate a semi-infinite medium.

(6) Hourglass control

The keyword *CONTROL_HOURGLASS is added to adjust and control the hourglass energy, and the keyword *CONTROL_BULK_VISCOSITY is added to adjust and control the quadratic and linear coefficients to complete the process of increasing the bulk viscosity in the material model.

In the simulation experiments, the *MAT_ADD_EROSION keyword was used to manage the damage of the samples during the simulation. Proper adjustment of the simulation parameters, maximum principal strain, and maximum principal stress can significantly improve the accuracy of the simulation results [54]. Based on this finding, the maximum principal strain and maximum principal stress were adopted as the judgement criteria for damage in this study, once the strain of the shale material exceeds the set value of the maximum principal strain or the stress exceeds the value of the maximum principal stress, the damage of the corresponding element will occur.

During the construction process of explosive fracturing, the lowest peak pressure of the initial well fracturing can be calculated according to the fracture pressure of this well and the explosive combustion model to provide a reasonable impact load design (as shown in Table 3) for explosive fracturing.

**Table 3:** Impact load material modelling parameters.

	Density /(g/cm^3^)	Dcj/(m/s)	Pcj/GPa	A/GPa	B/GPa	R1	R2	$E_{0}$/GPa	w
1	1.18	5500	10	276.2	8.44	5.1	2.1	3.87	0.5
2	1.59	7500	25	382.4	6.635	4.1	1.2	8	0.38
3	1.77	8500	35	634.7	7.998	4.2	1	8.9	0.3

## 4. Numerical simulation results and analysis of reservoir fracture propagation under impact load

### 4.1. Numerical simulation results and analysis of fracture propagation under different impact loads in homogeneous reservoirs

**Fig 14:**
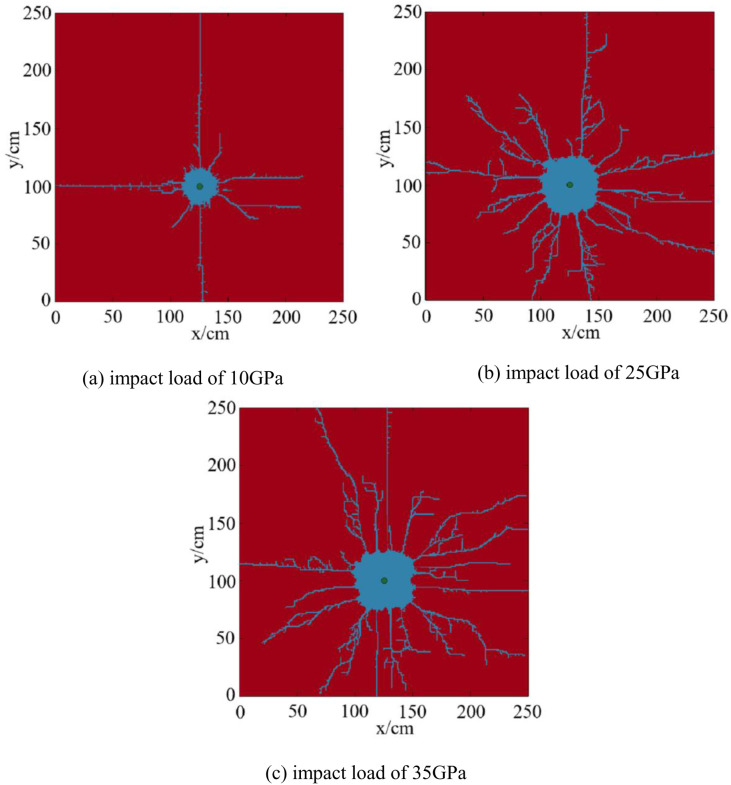
Numerical simulation results of fracture propagation under different impact loads in homogeneous reservoirs. (a) impact load of 10GPa, (b) impact load of 25GPa, (c) impact load of 35GPa.

The numerical simulation results of fracture propagation under different impact loads in homogeneous reservoirs are shown in Fig 14.

Under the impact load of 10GPa, eight main fractures and a large number of microfractures are produced. The proportion of the fracture distribution area is 7.09%. Under the impact load of 25 GPa,15 main fractures and a large number of microfractures are produced. The proportion of the fracture distribution area is 13.31%. Under the impact load of 35 GPa, 17 main fractures and a large number of microfine fractures are produced. The proportion of the fracture distribution area is 16.18%.

**Fig 15:**
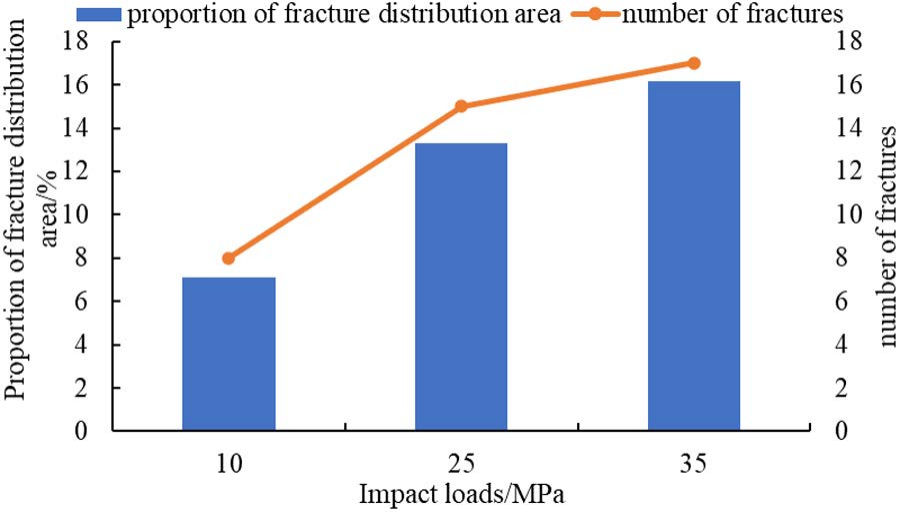
The curve of impact loads, proportion of fracture distribution area, and the number of fractures.

The curve of impact loads, the proportion of fracture distribution area, and the number of fractures are plotted, as shown in Fig 15.

Both the distribution area of fractures and the number of fractures increase with the increase of impact loads, as shown in Fig 15. This is because as the impact load increases, the stress becomes more concentrated. In areas of stress concentration, reservoir rocks are more prone to breakage, and the fracture situation is more complex, which leverages the advantages of explosive fracturing in reservoir modification and improves reservoir connectivity. This result is consistent with the conclusions of Wan et al [18] and Wang et al. [23], where the number of cracks increases with the increase in the loading rate.

### 4.2. Numerical simulation results and analysis of fracture propagation under different impact loads in reservoirs with a single layer

**Fig 16:**
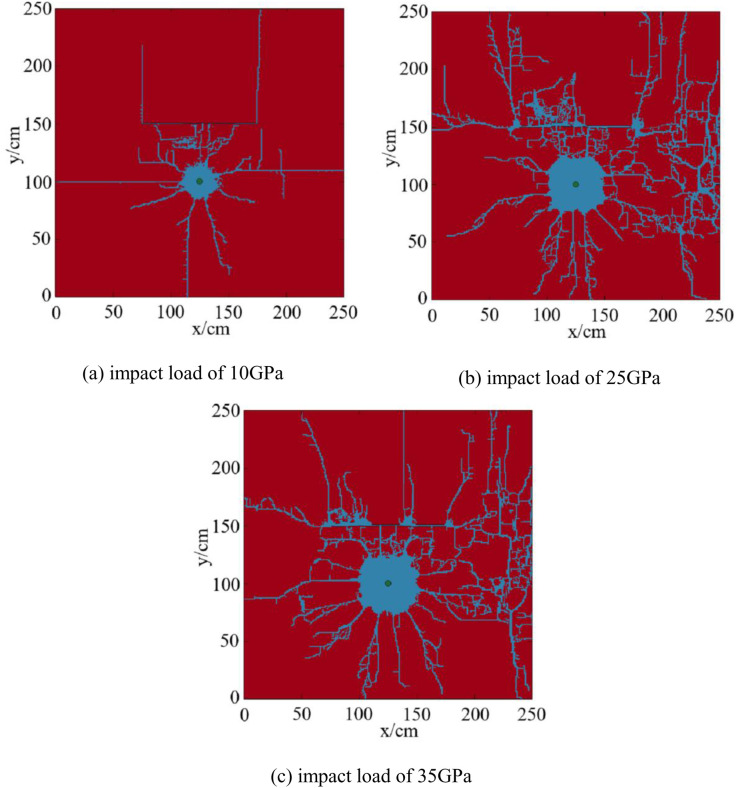
Numerical simulation results of fracture propagation under different impact loads in reservoirs with a single layer. (a) impact load of 10GPa, (b) impact load of 25GPa, (c) impact load of 35GPa.

The numerical simulation results of fracture propagation under different impact loads in reservoirs with a single layer are shown in Fig 16.

As can be seen from Fig 16, under the impact load of 10 GPa, 12 main fractures and a large number of microfractures are produced. The proportion of the fracture distribution area is 9.71%. Under the impact load of 25 GPa, 18 main fractures and a large number of micro-fractures are produced, and the proportion of fracture distribution area is 23.75%. Under the impact load of 35 GPa, 21 main fractures and a large number of microfractures are produced, and the proportion of fracture distribution area is 24.61%.

**Fig 17:**
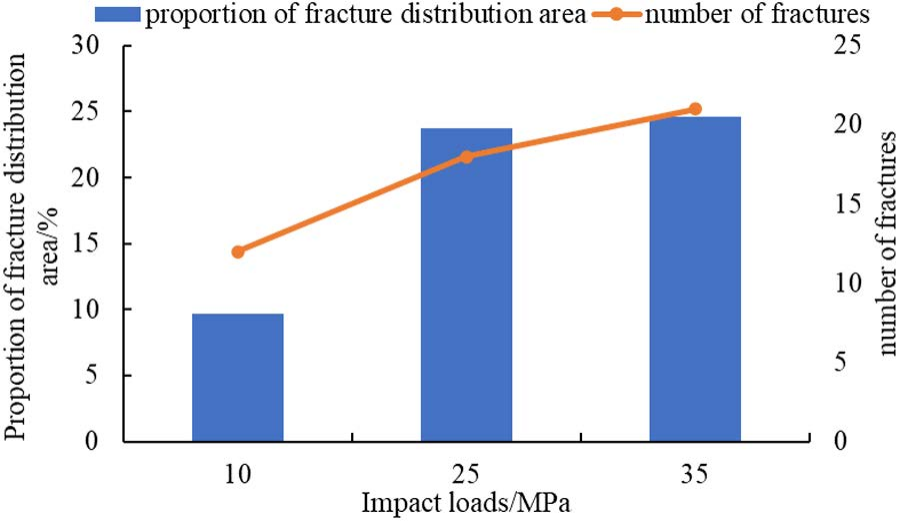
The curve of impact loads, proportion of fracture distribution area, and the number of fractures.

Plot the curve of impact loads, proportion of fracture distribution area, and the number of fractures (Fig 17).

The distribution area of fractures and the number of fractures increase with the increase of impact loads, as shown in Fig 17. This is because as the effective stress increases, when it exceeds the tensile strength of the rock, it can cause instantaneous fracture of the rock on the stressed surface. Compared with the homogeneous model, the influence between fractures in reservoirs with a single fracture is increased. The original fracture zone is more prone to inducing stress. The interaction of multiple fractures leads to the bending of the propagation path, making it easy to form a network of fractures. This will improve the diversion capacity of the original fracture zone, which is beneficial for the reservoir transformation effect of explosive fracturing.

### 4.3. Numerical simulation results and analysis of fracture propagation under different interlayer spacings in reservoirs with multiple layers

**Fig 18:**
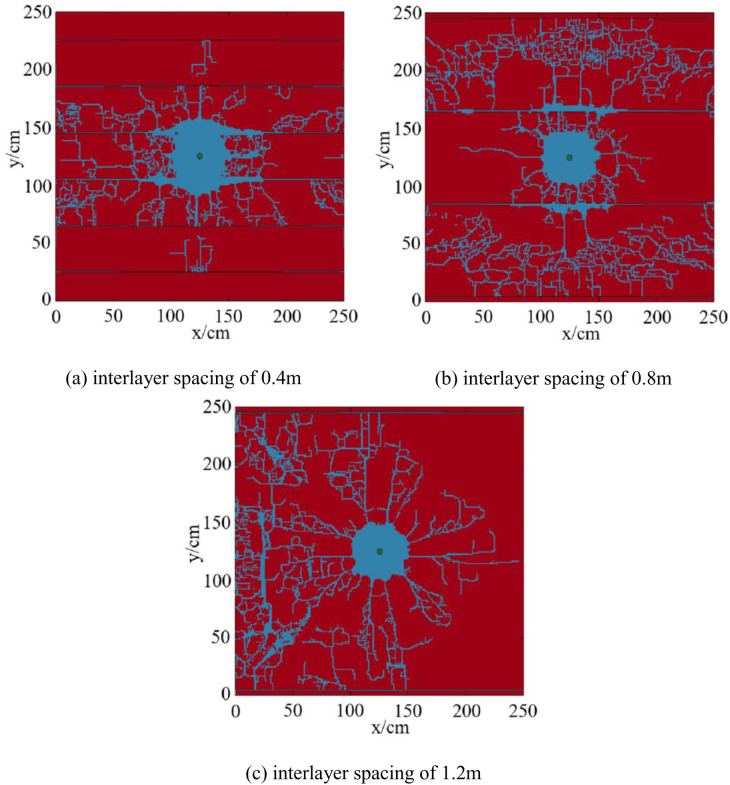
Numerical simulation results of fracture propagation under different interlayer spacings in reservoirs with multiple layers. (a) interlayer spacing of 0.4m, (b) interlayer spacing of 0.8m, (c) interlayer spacing of 1.2m.

The numerical simulation results of fracture propagation at different interlayer spacings under a 25GPa impact load in reservoirs with multiple layers are shown in Fig 18.

When the interlayer spacing is 0.4m, 25 main fractures and a large number of microfractures are produced. The proportion of the fracture distribution area is 17.37%. When the interlayer spacing is 0.8m, 23 main fractures and a large number of micro-fractures are produced, and the proportion of fracture distribution area is 24.73%. When the interlayer spacing is 1.2m, 13 main fractures and a large number of microfractures are produced, and the proportion of the fracture distribution area is 33.11%.

**Fig 19:**
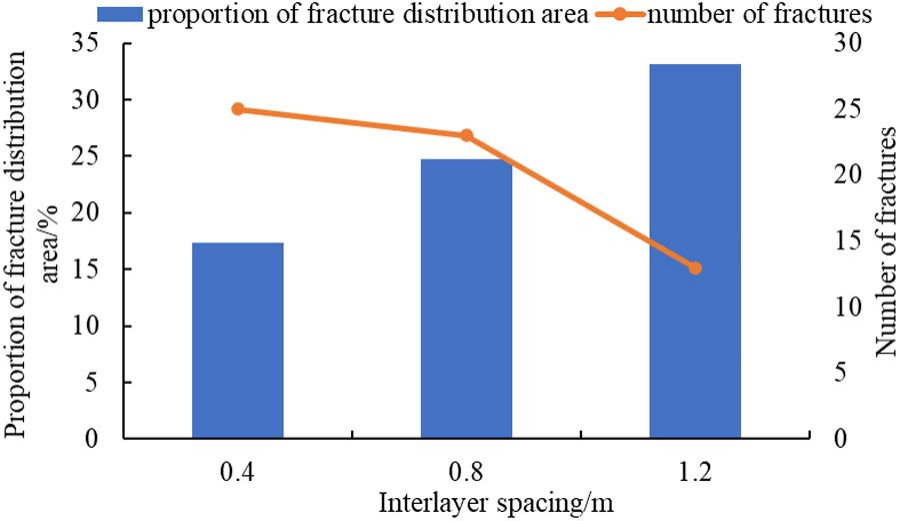
The curve of interlayer spacing, proportion of fracture distribution area, and the number of fractures.

Plot the curve of interlayer spacing, proportion of fracture distribution area, and the number of fractures (Fig 19).

As can be seen from Fig 18, the explosive stress wave propagates rapidly in the reservoir during the explosive fracturing process. This is because when the interlayer spacing decreases, the mutual influence of fractures intensifies. The near wellbore area is more prone to induced stress and the formation of network fractures, with a higher number of fractures. Fracturing in the near wellbore area is mainly caused by tensile failure, resulting in an increase in fracture width and a shorter extension distance. At this point, the damping of the shock wave propagation towards distant places increases, making it less likely for fractures to propagate to distant areas and making crack extension more difficult. When the interlayer spacing increases, the mutual influence of fractures becomes smaller, which is conducive to shear fracture. After the fractures generated by the impact load penetrate the layered structure, they will expand further away. The compression stress wave between reservoirs and the reflected tensile stress wave superimpose, forming a stress concentration zone. A large number of micro fractures have emerged in the reservoir rock at the far wellbore end, and the fracture extension distance is relatively far, which is more conducive to reservoir transformation in the far wellbore area. This result is similar to the research conclusion of Shen et al [27]. Their results reveal that the radial compressive stress of narrowly-spaced parallel joints is much higher than that of widely-spaced parallel joints. When the spacing is large, the position of the maximum radial compressive stress shifts, which is conducive to the development of blasting cracks in the middle. Meanwhile, Wang et al. [29]‘s study also pointed out that natural fractures are a negative factor for fracture propagation in methane deflagration fracturing, and the larger the number of natural fractures, the more significant the hindrance to the radial propagation of the deflagration fractures.

### 4.4. Numerical simulation results and analysis of fracture propagation under different impact loads in reservoirs with multiple layers

**Fig 20:**
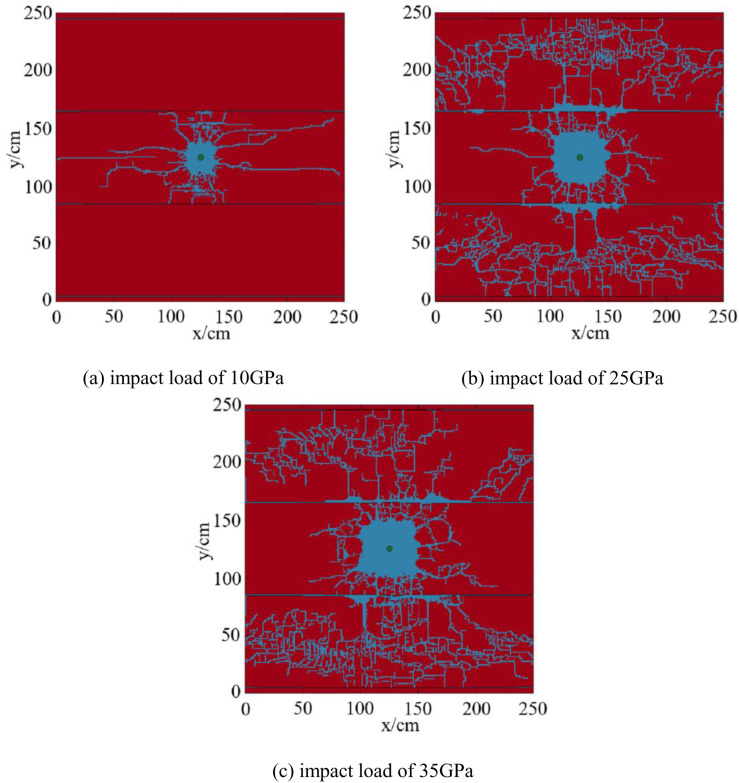
Numerical simulation results of fracture propagation under different impact loads in reservoirs with multiple layers. (a) impact load of 10GPa, (b) impact load of 25GPa, (c) impact load of 35GPa.

The magnitude of the blast impact load directly affects crack propagation [55]. Based on the single layer model, establish a fracture propagation model for a multiple layers reservoir under different impact loads, and analyze the influence of different impact loads on reservoir fracture propagation. The numerical simulation results of fracture propagation with the interlayer spacing of 0.8m are shown in Fig 20.

As can be seen from Fig 20, under the impact load of 10 GPa, 13 main fractures and a large number of microfractures are produced, which failed to penetrate the bedding layer. The proportion of the fracture distribution area is 5.52%. Under the impact load of 25 GPa, 21 main fractures and a large number of micro-fractures are produced, and the proportion of fracture distribution area is 24.73%. Under the impact load of 35 GPa, 28 main fractures and a large number of microfractures are produced, and the proportion of fracture distribution area is 25.64%. Under the impact loads of both 25GPa and 35GPa, the fractures penetrate the bedding layer.

**Fig 21:**
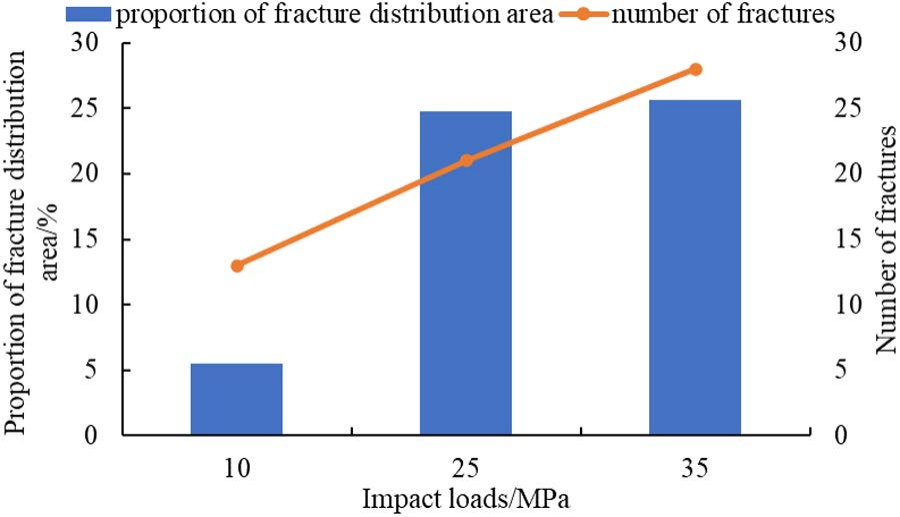
The curve of impact loads, proportion of fracture distribution area, and the number of fractures.

Plot the curve of impact loads, proportion of fracture distribution area, and the number of fractures (Fig 21).

As shown in Fig 21, the distribution area of fractures and the number of fractures increased with the increase of impact loads. This is consistent with the simulation results conducted by Wang et al [30]. on shale, which indicates that increasing explosive loading boosts fracture network development. Higher impact loads can penetrate layer fractures, causing shock waves to propagate further and causing greater damage to shale reservoirs. Although there were many cracks generated by impact loads, not all of them can extend, only a few of the main cracks can extend to the far wellbore area. By comparing the fracturing effects under different impact loads, it was found that increasing the impact load did not significantly increase the number of fractures near the wellbore, but the proportion of fracture distribution area was higher.

## 5. Conclusion

In order to further clarify the mechanism of shale fracture propagation under the impact load of explosive fracturing, the SHPB tests have been conducted, and a study on the fracture propagation law of shale reservoirs under impact loads has been conducted by using the finite element method. The following conclusions are mainly obtained:

(1) SPHB tests have been conducted. Comparing test data from three different impact velocities, it is found that the degree of sample pulverization increased with the increase of impact velocity. By analyzing the stress-strain curves at three impact velocities, the average dynamic elastic modulus of the shale reservoir has been determined to be 4.42 GPa.
(2) Based on test data, a homogeneous and isotropic crack propagation model has been established. Using finite element software ANSYS/LS-DYNA, the fracture propagation process under three different impact load conditions has been simulated. As the impact load increases from 10GPa to 35GPa, the crack situation becomes more complex in areas of stress concentration. The number of main cracks generated increases from 8 to 17, and the proportion of fracture distribution area increases from 7.09% to 16.18% (Fig 15).
(3) The laws of fracture propagation in shale reservoirs under different fracture distributions, interlayer spacing, and impact loads have been analyzed. The original crack zone of layered reservoirs is more prone to induce stress; the proportion of crack distribution area in the simulation results has increased from 9.71% to 24.61%(Fig 17), which is beneficial for reservoir transformation in the near wellbore area while explosive fracturing. The increase in interlayer spacing is beneficial for the expansion of the main crack, and the proportion of crack distribution area in the simulation results has increased from 17.37% to 33.11% (Fig.19), which can improve reservoir connectivity. As the impact load increases, the propagation range of shock waves expands, the proportion of crack distribution area in the simulation results has increased from 5.52% to 25.64% (Fig.21), and the main cracks have better connectivity.
